# The age factor in survival of a population cohort of well-differentiated thyroid cancer

**DOI:** 10.1530/EC-13-0056

**Published:** 2013-09-21

**Authors:** Andrea Mazurat, Andrea Torroni, Jane Hendrickson-Rebizant, Harbinder Benning, Richard W Nason, K Alok Pathak

**Affiliations:** Section of Surgical Oncology, Department of SurgeryCancerCare Manitoba, University of ManitobaGF440 A 820 Sherbrook Street, Winnipeg, Manitoba, R3A 1R9Canada

**Keywords:** risk stratification, thyroid carcinoma, prognosis, survival, outcome

## Abstract

Well-differentiated thyroid carcinoma (WDTC) represents a group of thyroid cancers with excellent prognosis. Age, a well-recognized risk factor for WDTC, has been consistently included in various prognostic scoring systems. An age threshold of 45 years is currently used by the American Joint Cancer Committee-TNM staging system for the risk stratification of patients. This study analyzes the relationship between the patients' age at diagnosis and thyroid cancer-specific survival in a population-based thyroid cancer cohort of 2115 consecutive patients with WDTC, diagnosed during 1970–2010, and evaluates the appropriateness of the currently used age threshold. Oncological outcomes of patients in terms of disease-specific survival (DSS) and disease-free survival (DFS) were calculated by the Kaplan–Meier method, while multivariable analysis was done by the Cox proportional hazard model and proportional hazards regression for sub-distribution of competing risks to assess the independent influence of various prognostic factors. The mean age of the patients was 47.3 years, 76.6% were female and 83.3% had papillary carcinoma. The median follow-up of the cohort was 122.4 months. The DSS and DFS were 95.4 and 92.8% at 10 years and 90.1 and 87.6% at 20 years, respectively. Multivariable analyses confirmed patient's age to be an independent risk factor adversely affecting the DSS but not the DFS. Distant metastasis, incomplete surgical resection, T3/T4 stages, Hürthle cell histology, and male gender were other independent prognostic determinants. The DSS was not independently influenced by age until the age of 55 years. An age threshold of 55 years is better than that of 45 years for risk stratification.

## Introduction

Well-differentiated thyroid carcinoma (WDTC) represents a group of thyroid cancers that are associated with increasing incidence and excellent posttreatment outcome (1). This group comprises different histological types, the most common being papillary carcinoma, followed by follicular carcinoma, and Hürthle cell variant of follicular carcinoma (2). Despite the high cure rates of WDTC, about 10% of the patients still die of this disease and an even larger proportion will develop loco-regional recurrence (2, 3, 4). Identification of factors associated with aggressive behavior in WDTC has been extensively pursued over the past four decades, yielding a multitude of risk stratification systems, which aim to differentiate high-risk and low-risk patients on the basis of the tumor and patient characteristics. Several factors have been associated with aggressive behavior in WDTC, including the age at diagnosis, the gender of the patient, the size and extension of the primary tumor, and the presence of distant metastases (1, 2, 3, 4, 5).

The age at diagnosis, in particular, is considered to be a strong prognostic indicator of WDTC outcome and has been consistently included in the majority of risk stratification systems currently used in clinical practice (6, 7, 8, 9, 10, 11, 12, 13, 14, 15, 16). Interestingly, the widely adopted American Joint Cancer Committee (AJCC)-TNM staging system (10) includes age as a prognostic determinant exclusively for WDTC, classifying patients younger than 45 as low risk, independent of the presence of other risk factors such as primary tumor extension (T) or nodal involvement (N). Other risk stratification systems such as AMES (9), Noguchi *et al*. (11), GAMES (15), National Thyroid Cancer Treatment Cooperative Study (NTCTCS) (12), UAB and MDA (8), and CIH (13) follow the scheme of TNM adopting an age threshold ranging from 41 to 51 years, to stratify the patients in high- and low-risk groups. A different set of prognostic scoring systems, namely EORTC (7), AGES (14), MACIS (16), SAG (6), Murcia (17), and Ankara (18), on the other hand, considers age as a continuous variable that affects the total prognostic score in a multiplicative fashion without any stratification.

Several studies have shown that the relationship between the age at diagnosis and the disease-specific survival (DSS) of WDTC is inversely proportional and is better represented by an exponential function, suggesting that age should preferably be considered as a continuous prognostic variable, rather than a categorical parameter (5, 19). The rationale for including an age threshold in many risk stratification systems is the need for a standard, simple, and practical method to predict oncological outcome rather than generating complicated prognostic scores. However, the selection of an appropriate age threshold is critical to this process and an inaccurate value can lead to either overtreatment of low-risk patients, or under-treatment of high-risk ones. The objective of this study was to analyze the relationship between the age at diagnosis and the oncological outcome of WDTC.

## Subjects and methods

This study was approved by the Research Ethics Board of the University of Manitoba. Our population-based historical cohort includes all 2125 consecutive well-differentiated thyroid cancers diagnosed in 2115 patients in the province of Manitoba, Canada, from January 1, 1970 to December 31, 2010. We reviewed the individual electronic and paper records of diagnosis and treatment for this cohort of patient registered in the Manitoba Cancer Registry at CancerCare Manitoba, the only comprehensive cancer center for a population of 1.2 million. The primary source of diagnostic information included 2007 pathology reports, 56 discharge summaries, 44 autopsy records, and eight operative reports. Patient demographics, the extent of their cancer at the initial presentation, the details of treatment modalities employed, pathology details, cancer recurrences during the follow-up, and the final outcome status as of January 1, 2013 were recorded. Clinically and radiologically detectable disease relapses were considered as recurrences and a cytology/biopsy confirmation was obtained for local/regional relapses whenever it was possible. For distant metastases, increased radioactive iodine (RAI) uptake with or without raised thyroglobulin was considered as evidence of recurrence. All patients who migrated out of the province (considered to be lost to follow-up) or died during the study period were censored at that point in time. Cause of death was obtained from autopsy records and death certificates, and individual patient records were reviewed to confirm exact cause of death and status of thyroid cancer at the time of death.

All cases were restaged according to the AJCC/Union Internationale Contre le Cancer (UICC) staging for thyroid cancer (7th Edition, 2009); the topography and the histology were re-coded by WHO International Classification of Diseases for Oncology (3rd Edition) codes and the disease, signs, and symptoms by International Classification of Diseases (10th Edition) codes to ensure uniformity. Additionally, as a part of our collaborative staging project, the pathology and treatment details of 683 (29.6%) cases were independently reviewed for accuracy.

### Statistical analysis

The data were managed and analyzed using SPSS for Windows version 20.0 (SPSS, IBM Corp., Armonk, NY, USA). Group means were compared by ANOVA and categorical data using the Pearson *χ*^2^ test with continuity correction, as appropriate. A *P* value <0.05 was considered to indicate statistical significance and 95% CIs were used to express reliability in the estimates. After checking for normality assumption, the mean and s.d. were used to express normally distributed data (such as the age of the patients) and median with interquartile range (IQR) were used for non-normally distributed data (such as the tumor size and the follow-up). The disease-free survival (DFS) and the DSS were estimated by the Kaplan–Meier product limit method, and the effect of age and other prognostic factors on DSS was assessed using the log rank test for pairwise comparison. The competing influence of other causes of mortality, such as death due to a second primary tumor or non-cancer deaths, was analyzed by multivariable proportional hazards regression for sub-distribution of competing risks using STATA version 12 (StataCorp., College Station, TX, USA). Multivariable analyses were also performed with Cox proportional hazard models to assess the independent effect of different age cutoffs on DSS after confirming the proportional hazard assumption.

## Results

The study cohort consisted of 1621 (76.6%) females and 494 (23.4%) males with a mean age of 47.3±17.1 years. In all, 1762 (83.3%) patients had papillary carcinoma, 268 (12.7%) had follicular carcinoma, and 85 (4%) had Hürthle cell carcinoma. Nine patients had a synchronous second primary tumor of a different histology along with a papillary thyroid cancer (follicular-3, Hürthle cell-3, medullary-2, poorly differentiated-1), while one patient had a metachronous second papillary carcinoma in the contralateral thyroid lobe, 25 years after initial management. The median tumor size was 20 mm (IQR=10–34 mm) and microcarcinoma (tumor size ≤10 mm) represented 26.1% of all WDTC. Multifocal thyroid cancers were observed in 600 (32.7%) cases and a gross extra-thyroidal extension of tumor in 379 (17.9%) cases. At the time of diagnosis, 472 (22.3%) patients had regional lymph node involvement and 54 (2.6%) had distant metastasis. On January 1, 2013, 1658 (78.5%) patients had no evidence of disease; 49 (2.3%) patients were alive with disease; 105 (5.0%) patients were dead because of thyroid cancer; 78 (3.7%) patients had died of a second primary tumor; and 225 (10.6%) patients died of other causes. Thyroid cancers, which were incidental autopsy findings in 44 (2.1%) patients, were excluded from survival analysis as they had no treatment or follow-up. Three (0.1%) patients who had a synchronous medullary or poorly differentiated carcinoma were excluded from further analysis as these synchronous malignancies were thought to be more aggressive than their papillary thyroid cancer. Exclusions also included 42 (2.0%) patients, who died before their treatment due to unrelated causes; 15 (0.7%) patients, who were not considered as suitable surgical candidates; 13 patients (0.6%), who did not consent to thyroidectomy; and 11 (0.5%) patients, who were followed for <36 months. A total or near total thyroidectomy was performed in 1079 (54.3%) of the remaining 1987 patients, and of these patients, 788 (73%) had post-total thyroidectomy adjuvant RAI.

During the median follow-up of 124.6 months (IQR=57.8–227.3 months), 78 (3.9%) patients had posttreatment residual disease and 185 (9.3%) had disease recurrence at least 6 months after a successful initial treatment. The recurrences were observed in the residual thyroid lobe or thyroid bed in 24 (1.2%) cases, in the central compartment of the neck in 59 (2.9%) cases, in the lateral compartment of the neck in 40 (2.0%) cases, at a distant site in 47 (2.4%) cases, and at multiple sites in 15 (0.8%) cases. Ten- and 20-year DFS were 90.1% (95% CI, 88.5–91.4%) and 87.6% (95% CI, 85.6–89.3%), respectively. On multivariable analysis by the Cox proportional hazard model, DFS was significantly influenced by distant metastasis (hazard ratio (HR)=7.40; 95% CI, 4.54–12.07; *P*<0.001), incomplete surgical resection (HR=6.18; 95% CI, 4.02–9.49, *P*<0.001), T stages – T4 (HR=2.87; 95% CI, 1.80–4.58, *P*<0.001) and T3 (HR=2.41; 95% CI, 1.72–3.37, *P*<0.001), Hürthle cell histology (HR=2.34; 95% CI, 1.41–3.89; *P*=0.001), lymph node involvement (HR=2.14; 95% CI, 1.57–2.91; *P*<0.001), and male gender (HR=1.49; 95% CI, 1.11–2.00; *P*=0.008). The DFS was independent of the histology of follicular carcinoma (*P*=0.712, NS), extent of thyroidectomy (*P*=0.136, NS), and age of the patient at diagnosis (*P*=0.846, NS). Age had a non-monotonic relationship with the relapse of thyroid cancer.

**Figure 1:**
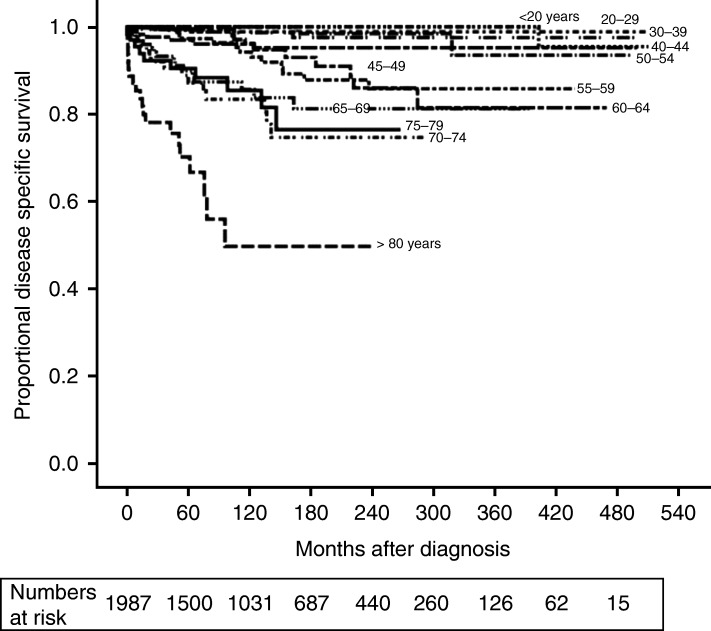
Disease-specific survival of well-differentiated thyroid cancer in different age groups.

The DSS was 95.4% (95% CI, 94.2–96.3%) at 10 years and 92.8% (95% CI, 91.2–94.2%) at 20 years. Twenty-year DSS was 99.0% (95% CI, 97.9–99.6%) for patients <45 years, 96.8% (95% CI, 93.1–98.5%) for patients in the age group of 45–54 years, 85.8% (95% CI, 78.7–90.7%) for patients in the age group of 55–64 years, and 74.0% (95% CI, 70.0–79.8%) for patients older than 65 years. Figure 1 shows no significant difference in the DSS in different age groups till the age of 55 years, when the first significant drop (*P*=0.002) was observed by log rank test for pairwise comparison. The case fatality rate of thyroid cancer also increased to 6.7% in the age group of 55–64 years from 2.2% in the age group of 45–54 years and thereafter it increased steadily with increasing age (Table 1).

**Figure 2:**
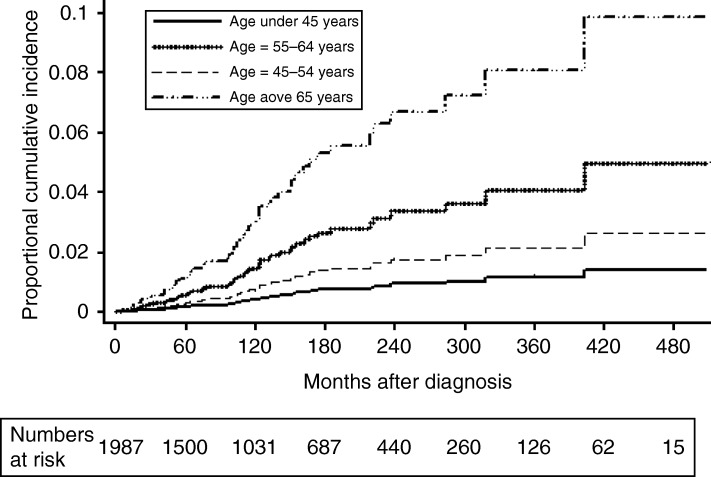
Cumulative incidence of death from thyroid cancer in different age groups by proportional sub-hazard model in competing risk analysis.

Cumulative incidences of death due to thyroid cancer at 10 and 20 years by multivariable competing risk analysis were 0.3 and 0.9% (age group <45 years), 0.7 and 1.8% (age group 45–54 years), 1.4 and 3.3% (age group 55–64 years), and 3.0 and 6.7% (age group >65 years), respectively (Fig. 2). There was an insignificant difference in the risks of dying from thyroid cancer between the age groups <45 and 45–54 years but the risk was significantly higher in the age groups 55–64 and >65 years. The risk of death from thyroid cancer was significantly influenced independently by the presence of distant metastasis (sub-hazard ratio (SHR)=12.65; 95% CI, 5.66–28.28; *P*<0.001), patient's age, i.e., 55–64 years (SHR=4.08; 95% CI, 1.52–10.92, *P*=0.005) or >65 years (SHR=5.72; 95% CI, 2.70–12.10, *P*<0.001), incomplete surgical resection (SHR=3.14; 95% CI, 1.52–6.47, *P*=0.002), T3/T4 stages (SHR=3.28; 95% CI, 1.74–6.17, *P*<0.001), Hürthle cell histology (SHR=4.67; 95% CI, 2.34–9.31; *P*<0.001), and male gender (SHR=2.08; 95% CI, 1.22–3.40; *P*=0.011). Patient's age, i.e., 45–54 years (*P*=0.154), histology of follicular carcinoma (*P*=0.204, NS), regional node involvement (*P*=0.07, NS), extent of thyroidectomy (*P*=0.136, NS), and adjuvant RAI (*P*=0.597) did not have any independent significant influence on the risk of death from thyroid cancer.

On multivariable analysis by the Cox proportional hazard model, the DSS was adversely influenced independently by age at diagnosis, distant metastasis, incomplete surgical resection, advanced T stage (T3/T4), Hürthle cell histology, and male gender (Table 2). The distribution of these prognostic factors in different age groups is summarized in Table 1. Lymph node involvement (*P*=0.07), histology of follicular carcinoma (*P*=0.110, NS), multifocality (*P*=0.956), extent of thyroidectomy (*P*=0.136), and adjuvant RAI (*P*=0.478) did not have a significant impact on the DSS of WDTC. There was an insignificant difference between the DSS of patients younger than 45 years and those in the age group of 45–54 years (*P*=0.141), but patients in the age group of 55–64 years had significantly higher risk of dying from thyroid cancer than those in the age group of 45–54 years (Table 2). On subset analysis of patients younger than 55 years and those 55 years and older, the advancing age was not an independent prognostic determinant of DSS in patients below 55 years (HR=1.03; 95% CI, 0.98–1.12; *P*=0.103); however, it had a significant impact on patients older than 55 years (HR=1.03; 95% CI, 1.01–1.09; *P*=0.01).

## Discussion

According to Canadian Cancer Statistics 2013, thyroid cancer has an excellent 5-year relative survival ratio of 98% (20). The relatively low proportion of adverse events and the long-term follow-up required to assess the outcomes of WDTC make a randomized clinical trial impractical to study these cancers. A large population-based cohort, such as ours, that is followed over a prolonged time period with a very low attrition rates (only 2.1% lost to follow-up over 40 years) is the most practical model to study these cancers. A cohort is a population group, or subset thereof, that is followed over a period of time. The members of the cohort, based on defined criteria, share common experience, which in this study was diagnosis of thyroid cancer in the province of Manitoba. By virtue of residence in the same province, our study cohort was expected to share a similar risk of exposure and get a similar standard of medical care in the publicly funded health care system of the province. The stability of the population cohort and a long, meticulous follow-up make the observations of this study very reliable.

A time span of four decades is both a strength and a limitation of our study as the diagnostic criteria, staging, and treatment recommendations have evolved over time. We uniformly restaged all cancers using the 2009 AJCC/UICC Cancer staging system for thyroid cancer and histology was re-coded by WHO International Classification of Diseases for Oncology (3rd Edition) codes to ensure consistency. In view of the indolent course of WDTC, it is possible that other causes of mortality, unrelated to thyroid cancer, could result in overestimation of thyroid cancer deaths by the Kaplan–Meier method. Consequently, we used competing risk regression to obtain an unbiased estimation of cumulative incidence of deaths resulting from thyroid cancer. Deaths due to second primary cancer and non-cancer deaths were treated as competing risks. We realize that the treatment recommendations for WDTC have also changed over the last four decades with total thyroidectomy and adjuvant RAI becoming more popular in the later part of the study (21), but neither had any significant influence on the DSS (Table 2). To maintain homogeneity of data across the study period, only clinically and radiologically detectable disease relapses were considered as recurrences. Isolated hyper-thyroglobulinaemia was not considered as evidence of recurrence.

Well-differentiated thyroid cancers, papillary, follicular, and Hürthle cell variants, account for over 90% of all thyroid malignancies (22, 23). Our analysis of all consecutive WDTC seen between 1970 and 2010 confirmed the excellent prognosis of WDTC with a DSS of 95.4% (95% CI, 94.2–96.3%) at 10 years. WDTC represents a group of unique cancers with the age of the patient at diagnosis being an important determinant of prognosis (Table 2). Although age has been consistently included as a risk factor in most of the risk stratification systems for WDTC, many of these (including the widely adopted TNM-AJCC) use age as a discrete categorical parameter for the ease of clinical staging. Nevertheless, there is a lack of consensus among the staging systems regarding the age threshold to be adopted. The AMES risk stratification system (9) recommends different age thresholds of 41 and 51 years for male and female patients, respectively, whereas Noguchi *et al*. (11), UAB and MDA (8), and CIH (13) risk stratification systems adopt an age threshold of 50 years. The TNM-AJCC staging system (10), along with the Memorial Sloan Kettering (15), and the NTCTCS (12) prognostic risk stratification systems use an age threshold of 45 years for WDTC patients independent of other prognostic parameters. Most of these recommendations are based on univariable analysis. In our study, the DSS for different age groups did not show a statistically significant decrease until the age of 55 years, when the first significant drop was observed (Fig. 1). This drop corresponds to an increase in the proportion of patients dying from thyroid cancer from 2.2 (45–54 years) to 6.7% (55–64 years) and an increase in the proportion of patients with T3/T4 tumors, Hürthle cell histology, incomplete resection, and distant metastasis (Table 1). We divided our study group into small age groups to find the most appropriate age threshold between 45 and 65 years. Even though the groups appear tiny, each group has close to 200 patients as the mean age of the cohort was 47.3 years. Once we arrived at the threshold of 55 years, we divided the cohort into bigger categories for multivariable comparison (Table 2 and Fig. 2). Multivariable analysis by the Cox proportional HR model and the competing risk analysis confirmed that the importance of age as an independent risk determinant becomes evident only after the age of 55 years (Fig. 2 and Table 2) and the subset analysis of patients above and below 55 years showed that the age was not an independent prognostic factor in patients below 55 years (*P*=0.103).

Age had a non-monotonic relationship with DFS and did not have an independent impact, which suggests that the recurrences in patients younger than 55 years are better salvaged. The currently practiced cutoff age of 45 years in TNM staging allocates patients between 45 and 55 years into a high-risk group and these patients may end up receiving unnecessary aggressive treatment even in the absence of other adverse prognostic factors such as distant metastasis, incomplete tumor resection, T3/T4 primary tumors, and Hürthle cell histology. However, it is interesting to see (Fig. 1) that the DSS of thyroid cancer continues to fall with a 3% reduction/year increase in age even beyond 55 years in the patients older than 55 years. A prognostic nomogram (24) incorporating the age and other prognostic factors may be a better tool to account for the variable influence of age in predicting the oncological outcome of thyroid cancer.

To conclude, our study confirms that patient age at the time of diagnosis is an independent risk factor with adverse impact on DSS of WDTC. The independent influence of age is not obvious till the age of 55 years. After the threshold age of 55 years, the DSS was found to fall in the older patients. An age threshold of 55 years is better than the threshold of 45 years currently used in the TNM staging.

## Author contribution statement

K A Pathak and R W Nason involved in study concept and design. A Mazurat, A Torroni, J Hendrickson-Rebizant, H Benning, R W Nason, and K A Pathak involved in data collection and analysis. A Mazurat, A Torroni, and K A Pathak involved in manuscript preparation. All authors reviewed the manuscript.

## Figures and Tables

**Table 1:** Distribution of various prognostic factors and deaths from thyroid cancer in different age groups. HCTC, Hürthle cell thyroid carcinoma.

**Age** (years)	**Males** (%)	**T3/T4 tumor** (%)	**HCTC** (%)	**Distant metastasis** (%)	**Incomplete resection** (%)	**Case fatality rate**
00–19 (*n*=84)	16.7	34.6	2.4	0	0	0 (0%)
20–29 (*n*=268)	19.0	21.5	0	0.7	2.4	1 (0.4%)
30–39 (*n*=413)	19.6	25.1	2.4	0.7	1.0	4 (1.0%)
40–44 (*n*=202)	21.3	19.7	4.5	1.5	1.6	4 (2.0%)
45–49 (*n*=212)	20.3	19.0	2.8	1.9	2.5	6 (2.8%)
50–54 (*n*=192)	22.9	23.6	5.7	0	1.7	3 (1.6%)
55–59 (*n*=193)	30.6	33.1	5.7	1.6	3.0	13 (6.7%)
60–64 (*n*=179)	30.7	34.0	6.1	4.5	5.1	12 (6.7%)
65–69 (*n*=133)	28.6	35.2	4.5	5.3	4.6	16 (12.0%)
70–74 (*n*=98)	32.7	32.5	6.1	8.2	8.4	15 (15.3%)
75–79 (*n*=73)	24.7	41.7	9.6	5.5	10.3	11 (15.1%)
80–89 (*n*=68)	23.5	53.8	8.8	17.6	14.0	20 (29.4%)

**Table 2:** Multivariable analysis by Cox proportional hazard model for independent influence of different prognostic factors on disease-specific survival.

**Prognostic factor**	**Hazard ratio** (95% CI)	***P* value**
Age at the time of diagnosis (years)		
<45	1.00 (reference)	
45–54	2.29 (0.76–6.89)	0.141 (NS)
55–64	5.62 (2.19–14.39)	<0.001
≥65	13.67 (5.78–35.38)	<0.001
Gender (male vs female)	2.13 (1.23–3.75)	0.007
T stage (early (T1–T2) vs advanced (T3–T4))	3.39 (1.79–6.43)	<0.001
Distant metastasis (M1 vs M0)	13.27 (6.47–27.21)	<0.001
Lymph node metastasis (N1 vs N0)	1.79 (0.96–3.33)	0.070 (NS)
Completeness of reaction (incomplete vs complete)	3.31 (1.70–6.43)	<0.001
Histology		
Papillary	1.00 (reference)	
Hürthle	4.40 (2.13–9.12)	0.001
Follicular	1.79 (0.88–3.68)	0.110 (NS)
